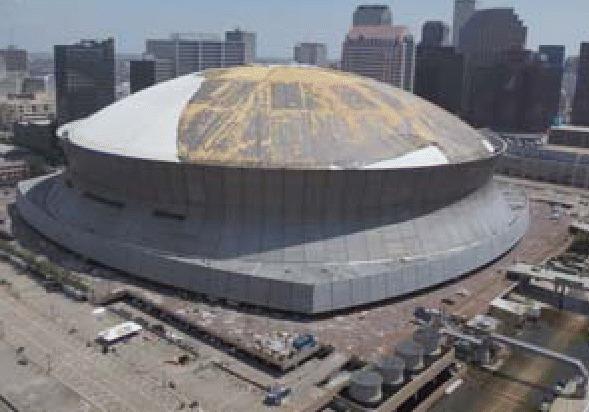# The Beat

**Published:** 2006-01

**Authors:** Erin E. Dooley

## Liver Library

Johnson & Johnson’s pharmaceutical research and development division has contributed a library of expression profiles for 100 paradigm compounds, primarily hepatotoxicants, to the Chemical Effects in Biological Systems (CEBS) knowledge base based at the National Center for Toxicogenomics, a part of the NIEHS. CEBS users can select arrays corresponding to one or more compounds from the library and use knowledge base tools to identify genes with significantly changed transcript levels. Lists of altered genes can then by annotated with current annotation provided by CEBS or projected onto biological pathways from groups like BioCarta, KEGG, and the Gene Ontology Consortium. CEBS is accessed at http://cebs.niehs.nih.gov/.

## Action for Indoor Air

At its 4 September 2005 congress, the International Academy of Indoor Air Sciences called on the governments, institutions, and corporations of the world to invest more in reducing indoor air pollution. According to the academy, indoor air pollution in developing countries can exceed international health-based guidelines by 20 times or more, and the use of coal contaminated with arsenic and fluorine is poisoning millions in China. The World Health Organization estimates that indoor solid fuel burning causes about 1.6 million premature deaths annually, mainly among women and children. These problems are easily solved, however. Low-cost interventions including education, improved cooking devices and fuels, better stove placement and ventilation, and a focus on reducing children’s exposures have been shown to successfully reduce the health effects of indoor air pollution.

## Nanodatabase Unveiled

The International Council on Nanotechnology and Rice University’s Center for Biological and Environmental Nanotechnology unveiled the world’s first database of scientific findings on nanotechnology on 19 August 2005. Available at http://icon.rice.edu/research.cfm, the database was created by Rice University researchers, the chemical industry, and the Department of Energy, and will be updated and enhanced over the next year. The database is searchable by author, year, keyword, type of particle, and type of experiment. Currently the database houses only abstracts and summaries of papers from peer-reviewed scientific journals, but policy reports and commentaries on key papers in the field will be added in the future.

## Arsenic in U.S. Rice

Researchers from Scotland’s University of Aberdeen reported in the 1 August 2005 issue of *Environmental Science & Technology* that U.S.-grown rice contains an average of 1.4 to 5.0 times more arsenic than from Europe, India, Bangladesh. Most U.S. rice is grown in fields that once grew cotton, which depends on arsenic-based chemicals to kill boll weevils and remove its leaves before harvesting. Because of the form that arsenic takes in the rice may not pose a threat; arsenic found in drinking water is estimated to be five times more toxic. However, one of the few epidemiological studies on eating a subsistence diet of arsenic-contaminated rice has linked it with an increase in bladder cancer.

## Managing Chemicals Together

Representatives of the world’s governments, intergovernmental groups, and other stakeholders met in Vienna in September 2005 to finalize the Strategic Approach to International Chemicals Management (SAICM). SAICM is a framework for global policy on chemical hazards and will ensure that by 2020 chemicals are manufactured and used in ways that minimize impacts on the environment and human health—a goal outlined at the 2002 World Summit on Sustainable Development. SAICM also promotes capacity building, technology transfer, and improved chemicals management, allowing better implementation of international treaties on chemicals such as the Basel Convention on the Transboundary Movement of Hazardous and Other Wastes. Three core documents from the Vienna meeting are expected to be adopted at a February 2006 conference in Dubai.

## Green Plan for Rebuilding NOLA

In the October 2005 issue of *Environmental Building News* (*EBN*), executive editor Alex Wilson outlines a 10-point plan for rebuilding New Orleans. The plan, developed with *EBN*’s editorial board and other sustainable planning and design experts, calls first for the formation of a Sustainable New Orleans planning task force. Coast and floodplain restoration is cited as the first priority. The plan also calls for salvaging and warehousing building materials, rebuilding a stronger levee system that is integrated into a perimeter park, mandating green building of both housing and commercial structures, creating more sustainable Gulf Coast fisheries, cleaning up the new brownfields using the greenest means, and partnering with industry to clean up factories in the region.

## Figures and Tables

**Figure:**
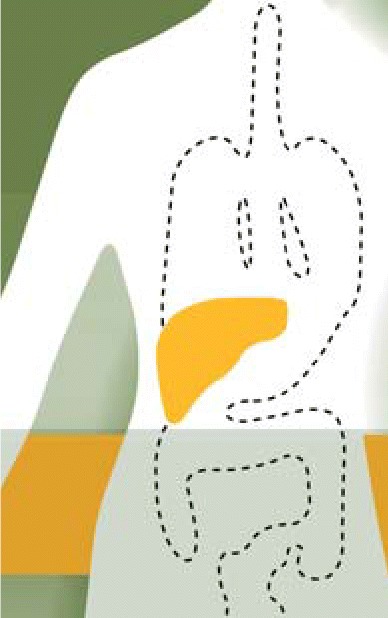


**Figure:**
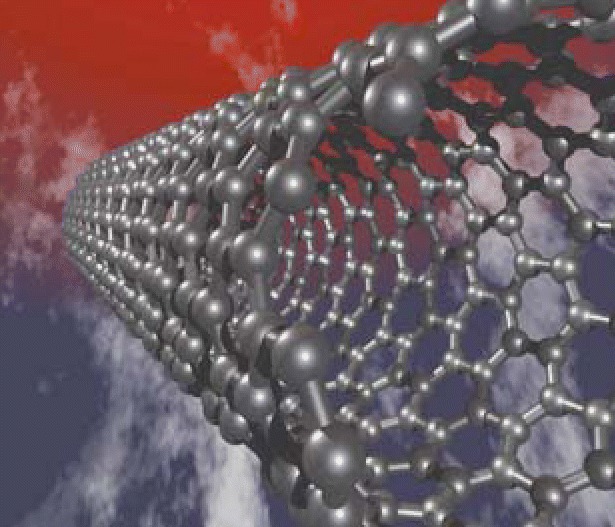


**Figure:**
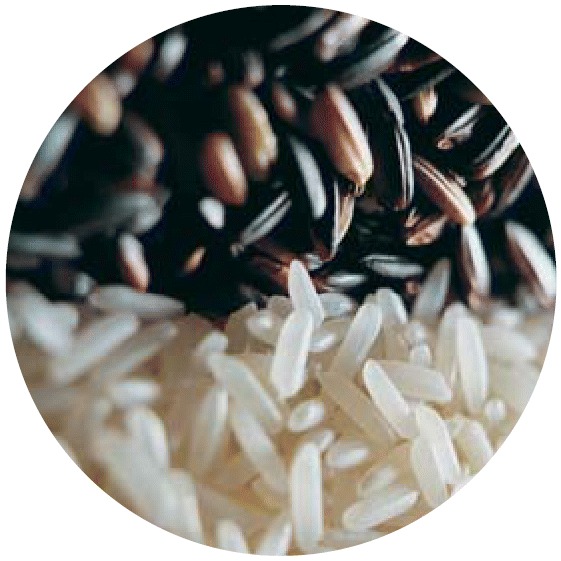


**Figure:**